# Supplementary material for: Nanobody-directed targeting of optogenetic tools to study signaling in the primary cilium
Source: eLife. 2020 Jun 24;9:e57907. doi: 10.7554/eLife.57907 (PMC7338050; doi:10.7554/eLife.57907)
Supplement: Supplementary file 1. — Plasmids are listed and the ID and sequence of the primers that have been used for cloning are indicated. [file elife-57907-supp1.docx]

**Supplementary File 1**

**Plasmids and cloning information.** Plasmids are listed and the ID and sequence of the primers that have been used for cloning are indicated.

| **pcDNA3.1_bPAC-mCherry** | | |
| --- | --- | --- |
| **ID** | **Sequence 5’-3’** | **Primer Info** |
| C0654 | CCGGATCCACCATGATGAAGCGGCTGGTGTAC | 5‘ primer for PCR bPAC, adds BamHI |
| C0631 | CCCAAGCTTGGCGCGCCGGCAGGCGCCACTTGGC | 3’ primer for PCR bPAC, adds AscI |
| C0633 | CCCAAGCTTACTTGTACAGCTCGTCCATG | 3‘ primer for PCR mCherry, adds HindIII |
| **pc6-LAPD-mCherry** | | |
| **ID** | **Sequence** | **Primer Info** |
| C2041 | GGATCCTAAGCTTCCACCATGAGCAGGGACCCCC | 5’ primer for PCR LAPD, adds HindIII/Kozak |
| C1724 | GGTTCTAGATTACTTGTACAGCTCGTCCATGCC | 3’ primer for PCR mCherry, adds XbaI |
| **pc3-mNphp3(201)-bPAC-mCherry** | | |
| **ID** | **Sequence 5’-3’** | **Primer Info** |
| C2956 | GGAGGATCCATGATGAAGCGGCTGGTGTAC | 5’ primer for PCR of mNPHP3, adds BamHI |
| C2954 | GTAGTCGGGCACGTCGTAGGGGTACTTGTACAGCTCGTCCATGCCG | 3’ primer for PCR mCherry, adds HA |
| C0609 | TCTTCTAGATTAGGCGTAGTCGGGCACGTCGTAGGGG | 3’ primer for PCR mCherry, adds HA, XbaI |
| **pc3-mNphp3(201)-LAPD-mCherry** | | |
| **ID** | **Sequence 5’-3’** | **Primer Info** |
| C2951 | GGAGGATCCATGAGCAGGGACCCCCTGCC | 5’ primer for PCR of mNPHP3, adds BamHI |
| C2952 | CACCATCGTCGCGACCGGTGGGTCCCGGGCCCGCGGTAC | 3’ primer for PCR of mNPHP3+linker, adds PVAT linker |
| C2953 | GACCCACCGGTCGCGACGATGGTG | 5’ primer for PCR of mNPHP3+linker, adds PVAT linker |
| C0609 | TCTTCTAGATTAGGCGTAGTCGGGCACGTCGTAGGGG | 3’ primer for PCR mCherry, adds HA, XbaI |
| **pcDNA3.1zeo_mCherry** | | |
| **ID** | **Sequence 5’-3’** | **Primer Info** |
| 5007 | GAAGGATCCACCATGGTGAGCAAGGGCGAGG | 5’ primer for PCR mCherry, adds BamHI |
| 5008 | CTCTCTAGATTACTTGTACAGCTCGTCCATG | 3’ primer for PCR mCherry, adds XbaI |
| **pc3-mNphp3(201)-mCherry** | | |
| **ID** | **Sequence 5’-3’** | **Primer Info** |
| C3037 | CGACGATGACGATAAGGATCCATGGTGAGCAAGGGCGAGG | 5’ primer for PCR of mNPHP3, adds BamHI |
| C2637 | ATATCTAGATTACTTGTACAGCTCGTCCATGCC | 3’ primer for mCherry, adds XbaI |
| **pcA-Cerulean** | | |
| **ID** | **Sequence 5’-3’** | **Primer Info** |
| 4037 | CGGGATCCACCATGGTGAGCAAGGGCGAG | 5’ primer for PCR of cerulean/citrine, adds BamHI |
| 4036 | GCTCTAGATTACTTGTACAGCTCGTCCATGCC | 3’ primer for PCR of cerulean/citrine, adds XbaI |
| **pcA-Citrine** | | |
| **ID** | **Sequence 5’-3’** | **Primer Info** |
| 4037 | CGGGATCCACCATGGTGAGCAAGGGCGAG | 5’ primer for PCR of cerulean/citrine, adds BamHI |
| 4036 | GCTCTAGATTACTTGTACAGCTCGTCCATGCC | 3’ primer for PCR of cerulean/citrine, adds XbaI |
| **pcDNA3.1-mNphp3(201)-VHH_LaM-2_-eGFP** | | |
| **ID** | **Sequence 5’-3’** | **Primer Info** |
| C4052 | ATTACTCGAG TGAGGAGACG GTGACCTGGG | 3' primer for PCR of VHH, XhoI in frame for fusion to eGFP |
| C4053 | TAATCTCGAG ATGGTGAGCA AGGGCGAGGA | 5' primer for PCR of eGFP, XhoI in frame for fusion to VHH |
| C4054 | ATTCTCTAGA TTACTTGTAC AGCTCGTCCA TGCC | 3' primer for PCR of eGFP, XbaI after Stop, for cloning eGFP to VHH |
| C4095 | TATAGGATCC CAGGTGCAGC TCGTGGAAAG TG | 5' primer for PCR of VHH-LaM-2, adds BamHI for fusion to mNPHP3 and adds Kozak |
| **pcDNA3.1-mNphp3(201)-VHH_LaM-2_-HA** | | |
| **ID** | **Sequence 5’-3’** | **Primer Info** |
| C4095 | TATAGGATCC CAGGTGCAGC TCGTGGAAAG TG | 5' primer for PCR of VHH-LaM-2, adds BamHI for fusion to mNPHP3 and adds Kozak |
| C4144 | GGTCTAGATC ACGCATAATC CGGCACATCA TACGGATATG AGGAGACGGT GACCTGG | 3' primer for PCR of VHH with HA tag at 3' end, adds Stop codon and XbaI for ligation into vector |
| **pcDNA3.1-VHH_LaM-2_-eGFP** | | |
| **ID** | **Sequence 5’-3’** | **Primer Info** |
| C4096 | TATAGGATCC ACCATGCAGG TGCAGCTCGT GGAAAGTG | 5' primer for PCR of VHH-Lam2, adds BamHI for cloning into vector, Kozak, ATG |
| C4052 | ATTACTCGAG TGAGGAGACG GTGACCTGGG | 3' primer for PCR of VHH, XhoI in frame for fusion to eGFP |
| C4053 | TAATCTCGAG ATGGTGAGCA AGGGCGAGGA | 5' primer for PCR of eGFP, XhoI in frame for fusion to VHH |
| C4054 | ATTCTCTAGA TTACTTGTAC AGCTCGTCCA TGCC | 3' primer for PCR of eGFP, XbaI after Stop, for cloning eGFP to VHH |
| **pcDNA3.1-mNphp3(201)-VHH_LaM-4_-eGFP** | | |
| **ID** | **Sequence 5’-3’** | **Primer Info** |
| C4051 | TATAGGATCC CAGGTGCAGC TCGTGGAATC TG | 5' primer for PCR of VHH, BamHI in frame for fusion to mNphp3(201) |
| C4052 | ATTACTCGAG TGAGGAGACG GTGACCTGGG | 3' primer for PCR of VHH, XhoI in frame for fusion to eGFP |
| C4053 | TAATCTCGAG ATGGTGAGCA AGGGCGAGGA | 5' primer for PCR of eGFP, XhoI in frame for fusion to VHH |
| C4054 | ATTCTCTAGA TTACTTGTAC AGCTCGTCCA TGCC | 3' primer for PCR of eGFP, XbaI after Stop, for cloning eGFP to VHH |
| **pcDNA3.1-mNphp3(201)-VHH_LaM-4_-HA** | | |
| **ID** | **Sequence 5’-3’** | **Primer Info** |
| C4051 | TATAGGATCC CAGGTGCAGC TCGTGGAATC TG | 5' primer for PCR of VHH, BamHI in frame for fusion to mNphp3(201) |
| C4144 | GGTCTAGATC ACGCATAATC CGGCACATCA TACGGATATG AGGAGACGGT GACCTGG | 3' primer for PCR of VHH with HA tag at 3' end, adds Stop codon and XbaI for ligation into vector |
| **pcDNA3.1-VHH_LaM-4_-eGFP** | | |
| **ID** | **Sequence 5’-3’** | **Primer Info** |
| C4052 | ATTACTCGAG TGAGGAGACG GTGACCTGGG | 3' primer for PCR of VHH, XhoI in frame for fusion to eGFP |
| C4053 | TAATCTCGAG ATGGTGAGCA AGGGCGAGGA | 5' primer for PCR of eGFP, XhoI in frame for fusion to VHH |
| C4054 | ATTCTCTAGA TTACTTGTAC AGCTCGTCCA TGCC | 3' primer for PCR of eGFP, XbaI after Stop, for cloning eGFP to VHH |
| C4061 | TATAGGATCC ACCATGCAGG TGCAGCTCGT GGAATCTG | 5' primer for PCR of VHH-LaM-4, adds BamHI for cloning into vector, Kozak, and ATG |
| **pcDNA3.1-mNphp3(201)-VHH_enhancer_-HA** | | |
| **ID** | **Sequence 5’-3’** | **Primer Info** |
| C4142 | AACGGATCCC AGGTGCAGCT GCAGGAATC | 5' primer for PCR of VHH-enhancer, adds BamHI for fusion to Nphp3 and deletes ATG |
| C4143 | GGTCTAGATC AGGCATAATC TGGGACATC | 3' primer cloning VHH-enhancer fusion with Nphp3, adds XbaI after HA and stop |
| **pc3.1-mNphp3(201)-VHH_enhancer_-mCherry** | | |
| **ID** | **Sequence 5’-3’** | **Primer Info** |
| C4142 | AACGGATCCC AGGTGCAGCT GCAGGAATC | 5' primer for PCR of VHH-enhancer, adds BamHI for fusion to Nphp3 and deletes ATG |
| C4263 | TAACTCGAGC ATCCCGGGTA CCATGCATCG | 3' primer for PCR of VHH-enhancer, deletes Stop and adds XhoI for fusion to mCherry |
| C4264 | GAACTCGAGG TGAGCAAGGG CGAGGAGGAT | 5' primer for PCR of mCherry, adds XhoI for fusion to VHH-enhancer and deletes ATG |
| C4265 | CGGTCTAGAT CACTTGTACA GCTCGTCCAT GC | 3' primer for PCR of mCherry, adds XbaI after Stop for ligation into vector |
| **pc3.1-VHH_enhancer_-mCherry** | | |
| **ID** | **Sequence 5’-3’** | **Primer Info** |
| C4265 | CGGTCTAGAT CACTTGTACA GCTCGTCCAT GC | 3' primer for PCR of mCherry, adds XbaI after Stop for ligation into vector |
| C4266 | TGAGGATCCA CCATGCAGGT GCAGCTGCAG GAATCGGG | 5' primer for PCR of VHH-enhancer-mCherry, adds BamHI for ligation into vector, Kozak, and ATG |
| **pEGFP-N1-bPAC** | | |
| C4443 | AATCGCTAGCCACCATGAAGCGGCTGGTGTACATC | 5' primer cloning bPAC in pEGFP-N1, adds NheI and Kozak before ATG |
| C4444 | GCCGAATTCTGTTCTTGTCGTTTTCCAGGGTCTGC | 3' primer cloning bPAC in pEGFP-N1, no stop, extra 4 bp, then EcoRI site |
